# Developing Interdisciplinary Training for Scientists in Cancer, Aging, and Disparities Research

**DOI:** 10.1093/geroni/igaf122.2537

**Published:** 2025-12-31

**Authors:** Jamie Mitchell, Elena Flores, Chiranjeev Dash, Lucile Adams-Campbell, Peter Lichtenberg, Jason Umans, Jeanne Mandelblatt

**Affiliations:** University of Michigan, Ann Arbor, Michigan, United States; University of Michigan, Ann Arbor, Michigan, United States; Georgetown University Lombardi Comprehensive Cancer Center, Washington, District of Columbia, United States; Georgetown University, Washington, District of Columbia, United States; Wayne State University, Detroit, Michigan, United States; Georgetown University, Washington, District of Columbia, United States; Georgetown University, Washington, District of Columbia, United States

## Abstract

The “Infrastructure for REsearch in Aging, Cancer and Health” (I-REACH) brings together a national coalition to increase the proportion of scientists conducting research at the crossroads of aging, disparities and cancer who are better prepared to improve the health of older cancer survivors. I-REACH developed an innovative and interdisciplinary eight module curriculum that integrates expertise across four academic hubs (Georgetown University, Wayne State/University of Michigan, UCLA, and University of Maryland). This expansive curriculum is designed to be hybrid in format, with pre-recorded lectures, live mentoring sessions, a resource library, and opportunities for early career scientists to present and receive feedback on their interdisciplinary work and pilot funding. In addition to a robust focus on cancer and aging biology, cognitive health, interventions, and social determinants of health, I-REACH also notably incorporates the lived experiences of older cancer survivors and caregivers throughout the curriculum. Scientists are mentored to conduct person-centered research that addresses stakeholder needs more effectively. Preliminary evaluation data demonstrates improvements in scholar research self-efficacy, collaborative partnerships, and grant development success. I-REACH offers a replicable model for training programs addressing complex health challenges requiring multidisciplinary approaches and meaningful stakeholder participation, with valuable insights for institutions developing similar initiatives.

